# Evolving Concepts in Our Understanding and Treatment of Holmes Tremor, Over 100 Years in the Making

**DOI:** 10.5334/tohm.683

**Published:** 2022-05-26

**Authors:** Grace Hey, Wei Hu, Joshua Wong, Takashi Tsuboi, Matthew R. Burns, Adolfo Ramirez-Zamora

**Affiliations:** 1 Norman Fixel Institute for Neurological Diseases, University of Florida, Gainesville, FL, US; 2 Department of Neurology, Nagoya University Graduate School of Medicine, Nagoya, JP

**Keywords:** Holmes tremor, midbrain tremor, rubral tremor

## Abstract

Holmes Tremor (HT) is an irregular, slow-frequency (<4.5 Hz) tremor characterized by a combination of resting, postural, and action tremors mostly of the upper extremities. Symptoms of HT typically emerge 4 weeks to 2 years after a brain injury caused by a spectrum of etiologies. HT pathophysiology is thought to result from aberrant collateral axonal sprouting and synaptic dysfunction following neuronal damage. To date, the dopaminergic nigrostriatal system, cerebello-thalamo-cortical pathway, and dentate-rubro-olivary pathway have all been implicated in the clinical manifestations of HT. The diversity of HT etiologies usually requires a personalized treatment plan. Current treatment options include carbidopa-levodopa, levetiracetam, and trihexyphenidyl, and surgical management such as deep brain stimulation in selected medication-refractory patients. In this review we discuss the pathophysiology, etiology, neuroimaging, and the latest clinical guidelines for care and management of HT.

## Introduction

Tremor is defined as an involuntary, rhythmic, or oscillatory movement in one or more body parts. It is one of the most common clinical presentations to movement disorders clinics [1]. Tremor can be broadly divided into rest or action, but tremors displaying postural, positional, or a combination of these components are also observed. Holmes Tremor (HT) was originally described in 1904 by Dr. Gordon Morgan Holmes as an irregular, slow-frequency (<4.5 Hz) tremor that may include one or a combination of resting, postural, and action tremors [2,3]. HT primarily affects proximal limbs, and often arises because of lesions located in the upper brainstem, thalamus, and cerebellum [3,4]. Lesions specifically involving the cerebello-thalamo-cortical and dentato-rubro-olivary pathways may contribute to the development of HT [4]. HT is reportedly caused by a diverse set of etiologies including traumatic injuries, infections, demyelination, and ischemic or hemorrhagic cerebrovascular disorders.

Given that multiple central nervous system (CNS) networks and structures are involved, HT is commonly associated with other clinical features including bradykinesia, spasticity, ataxia, and ophthalmoplegia [3]. HT symptoms typically begin 4 weeks to 2 years following neurological injury [3,4,5]. The delay of symptom onset is attributed to the aberrant synaptic rearrangements and deviant sprouting of collateral axons that pathologically occur over a period of time after neurons have been damaged [3,6]. Normally, damaged synapses are degenerated, reinnervated, and rearranged to restore healthy neuronal connections. In HT, it is thought that neuronal connections either become restored in a disorganized way or that connectivity between restored neurons is diminished, leading to reduced neurotransmitter release. However, the full mechanisms accounting for the delay in the onset of HT symptoms are not fully understood [5,9].

For this review, we searched PubMed for articles that describe HT characteristics. To focus our search on relevant publications we used the keywords “holmes tremor”, “rubral tremor”, “midbrain tremor”, OR “deep brain stimulation” AND “tremor”. These publications were primarily case reports and case series, and no randomized controlled trials were identified. We discuss the existing literature reporting the phenomenology, pathophysiology, etiologies, neuroimaging modalities, and available treatments for HT.

## Phenomenology and Historical Notes

In 1904, Gordon Holmes published “On Certain Tremors in Organic Cerebral Brain Lesions.” Although the reported cases varied with respect to localization of cerebral injury and clinical characteristics, each case demonstrated a characteristic irregular slow-frequency tremor varying from 3 to 5 oscillations per second appearing weeks after the initial injury [7]. Furthermore, each case demonstrated worsening tremor with agitation, excitement, or attempts to inhibit movement, and the absence of tremor during sleep [7]. Additional commonly reported and observed symptoms included weakness, diplopia, severe headache, and vomiting [7]. Individual cases of sensory disturbances, rigidity, weakness, impaired coordination, and changes in reflexes were reported although not common to every patient [7]. Holmes identified patients of all ages who suffered from falls, tumors, sudden “uselessness” of limbs, and tubercular joint disease, but without evidence of head injury prior to demonstrating symptoms of HT [7]. Furthermore, Holmes described cases where symptoms were sudden in onset and thus likely due to disease and lesions of vascular origin [7]. Holmes additionally highlighted the potential involvement of the cerebello-rubro-spinal system in HT and discussed the commonalities that HT shared with other types of tremor disorders, as well as the diverse range of etiologies and manifestations of HT in patients [7]. Holmes diagnosed all patients with midbrain lesions (leading to the traditional description as “midbrain tremors”), confirmed by postmortem evaluation and dissection [7].

The current definition of HT derives from criteria set by the Movement Disorder Society in 1998 recently updated in the Consensus Statement on the Classification of Tremors of the MD Society in 2018 [8]. In their review, and largely consistent with Holmes’ pioneering description, HT is a syndrome of rest, postural, and intention tremor that usually emerges from proximal and distal rhythmic muscle contractions at low frequency (<5 Hz) [8]. There is a variable delay between lesion occurrence or brain damage and tremor appearance, and slow frequency less than 4.5 Hz [2,6]. Other terms used to describe HT include rubral, thalamic, midbrain, or mesencephalic tremor. These terms, however, can be misleading because causative lesions or injuries localize to multiple cortical and subcortical brain areas. Thus, HT remains the preferred term [4,9]. A recent study identified only 155 patients reported from 1904 to 2016, suggesting that this tremor syndrome is rare [2].

## Etiology

**Figure 1:**
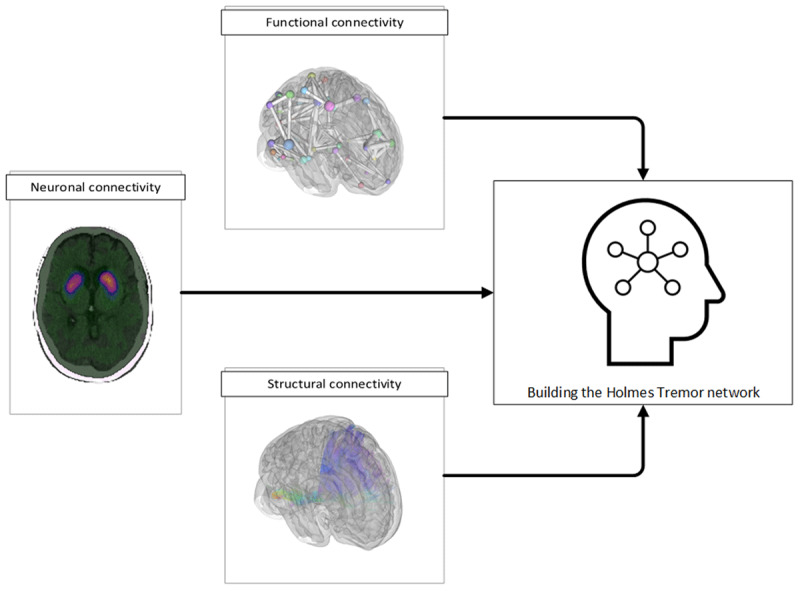
Neuroimaging advances in Holmes Tremor. Emerging multi-modal imaging strategies are increasingly being utilized to understand the underlying pathologic network in Holmes Tremor. These methods include functional MRI based functional connectivity, diffusion MRI based structural connectivity, and nuclear medicine imaging based neuronal connectivity.

In general, it is thought that multiple encephalic lesions affecting the dopaminergic nigrostriatal system, cerebello-thalamo-cortical pathway, and dentate-rubro-olivary pathway are required for the development and progression of HT [2,10]. Most patients with HT have lesions in the thalamus or midbrain [10,11]. The mechanisms of injury to these pathways include ischemic or hemorrhagic vascular lesions as well as traumatic brain injury, although the development and progression of HT can be linked to demyelinating diseases, metastatic cancer, paraneoplastic disorders, environmental toxins, and viral infections [2,11,12,13,14] Specifically, ischemic or hemorrhagic vascular lesions represent approximately 48–55% of HT cases, while instances of traumatic brain injury make up approximately 17–20% of cases [2,10]. Some studies found that hemorrhage resulting from traumatic brain injury is a common factor in the development of HT [3]. One recent report reviewed the etiologies of 29 cases of HT, 11 of which had hemorrhage as the primary cause of HT [2]. These data emphasize the importance of multiple injuries in the development of HT [2,3,9]. This same report showed no correlation between the extent of the lesions and the number and severity of associated neurologic manifestations [2]. This suggests that our ability to anatomically localize affected pathways using current neuroimaging is limited, and that we need a better understanding of functional connectivity and injury (***Figure 1***).

Other conditions have been associated with the occurrence of HT. It is proposed that viral or parasitic infections, including human immunodeficiency virus (HIV) and toxoplasmosis, can cause HT. It is thought however, that in these cases HT results from vasculopathy rather than structural lesions associated with infection [14]. Consistent with cases describing other etiologies, the unifying feature of all reported cases of HT in AIDS patients with CNS toxoplasmosis is the involvement of either the cerebello-thalamo-cortical and/or the dentato-rubro-olivary pathways. A required role for the nigrostriatal system, however, is less clear [15]. Although these findings reinforce the importance of both the cerebello-thalamo-cortical and the dentato-rubro-olivary pathways in the development of HT, some cases lacked involvement of the nigrostriatal system [15]. Additional imaging studies support normal dopaminergic uptake in certain cases [12,13,14,15]. It should be noted however, that this occurrence is uncommon as evidenced by being reported primarily in individual case reports [3,14,15]. Contrary to these cases, the development of HT following environmental carbon monoxide exposure leading to basal ganglia injury has been reported [12].

Genetic etiologies have been implicated in HT, such as the novel PNPLA6 mutations with cerebellar degeneration [11]. PNPLA6 is a critical gene responsible for producing membrane protein neuropathy target esterase (NTE), which aids in developing the embryonic neural tube. Mutations in this gene have been implicated in a wide range of neurodegenerative disorders [11]. In one case series, three Turkish sisters homozygous for the PNPLA6 mutation with non-consanguineous, unaffected, heterozygous carrier parents developed HT in a similar pattern [11]. Each sister demonstrated gait disturbances during childhood, and developed HT between the ages of 20 and 30 [11].

Further attempts to evaluate these network-level pathophysiologic changes have been pursued with neuroimaging techniques. HT lesions do not localize to one specific region, but rather are localized to functionally linked brain circuits. There is conflicting evidence, however, about brain lesion severity, size, timing, and HT-associated neurological manifestations. It is common for patients with HT to experience relatively severe and widespread lesions secondary to trauma or pre-existing genetic disorders resulting in enhanced or more widespread manifestations of HT due to the size of the injury [2]. However, other studies report no significant correlations between lesion extent and the severity of associated neurologic manifestations [2]. As previously stated, this discrepancy extends to neuroimaging, with occasional dissociation between symptoms severity and degree of injury on MRI [12,13,14,15]. The latency from lesion to tremor onset is usually about two months and its generation likely relies on a combined involvement of both the dopaminergic nigrostriatal system and the cerebello-thalamo-cortical pathways. Delayed tremor onset may suggest a secondary neural degeneration along the dentate-rubro-olivary pathways. Following brain damage, compensatory collateral sprouting and synaptogenesis is observed in the dopaminergic nigrostriatal system, cerebello-thalamo-cortical pathway, and dentate-rubro-olivary pathway of HT patients [2,3,4,5]. Which may result in aberrant synapse formation and reinnervation, leading to HT development.

## Neuroimaging Modalities

Just as there is no singular etiology behind HT, there is no single MRI sequence optimized to detect and identify all possible lesions [5]. As such, a comprehensive brain MRI that includes T1-weighted, T2-weighted and susceptibility weighted sequences should be performed. [5,6,10,14]. Localization of HT to a single neuroanatomical location (or even network) via imaging remains elusive, possibly due in part to the distributed nature of the pathologic systems that contribute to tremor [16]. Recent imaging studies have applied a lesion network mapping strategy to case reports of HT [17,18]. In these studies, the authors analyzed 36 case reports including structural brain scans identifying the casual lesion and constructed a “lesion connectome” using group-averaged resting state functional MRI (rs-fMRI) data from 1,000 healthy volunteers as part of the Brain Genomics Superstruct Project [19]. A common brain network among the HT cases was identified and includes the following nodes: the red nucleus, the globus pallidus interna (GPi), the ventral oralis posterior (VOP) nucleus, the pulvinar nucleus, the pontomedullary junction, the cerebellar cortex, the cerebellar vermis in lobule VI, and cerebellar cortex in lobule X. This HT lesion connectome was tested against cases that reported neurosurgical intervention data and observed that proximity to the connectome was potentially associated with the extent of symptomatic response.

The lesion connectome hypothesis was further explored by an fMRI study that concurrently acquired tremor accelerometry data and task-based fMRI data from the sensorimotor cortex and cerebellar vermis in a single patient with HT [20]. The resulting network was then compared to the previously proposed lesion connectome [17)]. The authors found that the tremor-related signal represented a distinct structural network but was functionally coupled with the lesion connectome. Nieuwhof and colleagues proposed that the underlying pathophysiology of HT may involve three distinct components: 1) a structural lesion 2) a correlative lesion connectome and 3) a tremor amplitude-related network.

A multi-modal imaging approach has also been applied to further evaluate dopaminergic network involvement specifically [2,21]. For example, single photon emission computed tomography (SPECT) imaging uses nuclear imaging technology to visualize blood flow to various parts of the brain and can be particularly helpful with subcortical structures [22]. One such radiotracer, I-123-ioflupane commercially known as DaTSCAN, can be used to determine dopaminergic striatal deficit in HT and potential levodopa responsiveness [2]. However, nuclear imaging results in the HT literature have been highly variable and challenging to interpret [23]. As Gajos and colleagues report in their study of 10 HT patients, there were no significant hemispheric differences in DaTSCAN dopamine uptake based on a visual assessment or quantitative measurements [24]. The authors proposed that since levodopa responsiveness can be interpreted as nigrostriatal involvement in HT, identification of dopaminergic deficit via nuclear imaging may have potential predictive value. This had been reported previously by a PET study that evaluated 6 HT patients with both fluorine-18-dopa (FDOPA) PET and bromide-76-lisuride (bromide-76) PET. FDOPA is another radiotracer that can measure striatal dopaminergic uptake while bromide-76 PET is a selective marker for the dopamine D2 receptor [25]. Other reports indicate that HT patients have significantly decreased dopamine uptake in the striatum ipsilateral to the lesion when compared to the contralateral striatum in controls. Bromide-76 PET revealed no differences, however, in ipsilateral D2 receptor concentration when compared to the contralateral hemisphere and both control groups, suggesting no D2 receptor upregulation despite the dopaminergic deficit. Interestingly, despite a lack of predictive imaging findings, all 6 patients reported experiencing improvement in rest tremor with carbidopa-levodopa. The authors proposed that multiple pathways may be contributing to the complex HT phenomenology. The rest tremor component may be related to dopaminergic dysfunction as highlighted by the FDOPA findings and levodopa responsiveness. However, the action and intention tremor components may be non-dopaminergic in nature and related to red nucleus or superior cerebellar peduncle pathology.

## Treatment

A variety of interventions have been explored for management of severe HT symptoms. The prominent amplitude and proximal limb involvement associated with HT regularly leads to marked difficulties with daily activities and fine motor tasks. There is a lack of controlled, clinical studies for treatment in HT, with most of the current therapeutic options based on small case series. Several medications, including carbidopa-levodopa, levetiracetam, trihexyphenidyl, dopamine agonists, and anticholinergics are commonly used, based on their efficacy in management of other tremor syndromes such as essential tremor (ET) and Parkinson’s disease (PD) [5,9]. HT patients, however, often exhibit highly variable responses to medical therapy [2,11,12,20]. Multiple studies suggest that carbidopa-levodopa is effective in approximately 50% of HT patients. [2,5,10]. A recent systematic review by our group found that medications, including levodopa, trihexyphenidyl, and levetiracetam can markedly improve tremor in HT [26]. Some reports suggest that HT can improve with dopaminergic agents even in the absence of clear nigrostriatal pathway damage. It is plausible that the nigrostriatal pathway is indirectly involved in HT development or is secondarily damaged by a primary lesion. Well-designed longitudinal neuroimaging longitudinal studies would be required to address this question further. Moreover, additional pharmacological agents including clonazepam, bromocriptine, amantadine, biperiden, or botulinum toxin injections can be considered as second line agents [26]. In patients with CNS toxoplasmosis, significant improvement of HT has been reported with antitoxoplasmic/steroid treatment, suggesting that direct treatment of the etiology is critical and may possibly reverse network dysfunction prior to durable neuronal loss [15].

When medication therapies fail or are inadequate, lesional surgery or neuromodulation can be considered but surgical outcomes are variable depending on the anatomic location of the lesions and neurological pathology (***Table 1***) [3]. Surgery has been explored with good outcomes using either stereotactic surgical ablation of the thalamic ventralis intermedius nucleus (Vim), pallidotomy, or with deep brain stimulation (DBS). There is an increased risk of side effects by placement of larger lesions with lesional surgery [17]. Different targets have been explored for the treatment of HT including the thalamic ventralis intermedius nucleus (VIM), globus pallidus internus (GPi), and subthalamic nucleus (STN) [9,11,17]. Although Vim is the most frequently used target owing to significant tremor suppression in other syndromes, several reports also suggest that Vim DBS may fail to improve tremor enough to reduce a patient’s disability. There are concerns related to tremor recurrence over time, and a limited effect on proximal or the intentional component [27].

**Table 1:** DBS outcomes for patients with HT in the literature.


AUTHORS	PATIENTS AND ETIOLOGIES	DBS TARGETS	OUTCOMES	FOLLOW-UPS

Bargiotas et al. (2021)	Four patients (cerebellar stroke, stroke of the left lateral thalamus and the internal capsule, mesencephalic hemorrhage due to a cavernous malformation, or pontomesencephalic/thalamic strokes)	VIM or DRTT	34–61% improvement in total TRS. Tremor improvement was lost in three years in three patients but sustained up to nine years in one patient.	Mean length of followup: 5 years

Cenzato et al. (2021)	Three patients who underwent resection of brainstem cavernomas	VIM for one patient, not described for two patients	Complete or almost complete tremor regression in all cases	N/A

Parker et al. (2021)	Hematoma in the right midbrain and cerebral peduncle	Left VIM + GPi	TRS 70% reduction in tremor	10 years

Ghanchi et al. (2020)	Immune reconstitution inflammatory syndrome due to highlyactive antiretroviral therapy for HIV infection (symptoms for 6 months)	Left VIM	Significant tremor improvement in the right arm	6 months

Razmkon et al. (2020)	Posttraumatic HT (symptoms for 6 years)	Right VIM	The patient experienced complete tremor suppression after DBS but developed localized infection. A rescue lesion through the implanted lead before explanting the DBS system controlled tremor well	3 years

O’Shea et al. (2020)	Right medial cerebral peduncle and bilateral thalamic strokes (symptoms for 2 years)	Right Vim + Zi	Clear improvement in tremors bilaterally	N/A

Dec-wiek et al. (2019)	Three patients (one with multiple sclerosis and two with ischemic stroke) (symptoms for 1–39 years)	PSA	TRS 56% reduction in tremor	1 year

Morishita et al. (2019)	Two patients (one with stroke and one with severe head trauma) (symptoms for 3 years)	VIM	TRS motor 52% reduction	6 months

Yuk et al. (2019)	Brainstem hemorrhage (symptoms for 3 months)	Left VIM	TRS part A 75% reduction	3 years

Martinez et al. (2018)	HIV-related vasculopathy associated with toxoplasmosis	Right Raprl	Robust and stable improvement in tremor	2 years

Aydın et al. (2017)	Posttraumatic HT	Left VIM + GPi	TRS > 80% tremor improvement	6 months

Toda et al. (2017)	Posttraumatic HT	VO + subthalamic region	While individual stimulation of each target was ineffective, an interleaved dual stimulation of both targets was effective	6 years

Ramirez-Zamora et al. (2016)	Pontine and midbrain hemorrhage secondary to rupture of arteriovenous fistula (symptoms for 7 years)	Left GPi	80% improvement in TRS right hand score	6 months

Kilbane et al. (2015) [17]	1. Right brainstem hemorrhage due to cavernous malformation 2.Multicystic brainstem tegmentum lesions 3.Left thalamic midbrain bullet fragment 4.Right thalamic/subthalamic infarction	Patient 1 had VIM/VOA and Gpi leads. Patient 2 and 4 had unilateral Gpi. Patient 3 had VIM/Gpi	TRS improved from a mean 53.25 points prior to surgery to 11.25% representing a 78.87% benefit	Mean length of followup: 33.7 months.

Espinoza-Martinez et al. (2015) [41]	Two patients with ICH due to cavernous malformations, four patients with cerebral infarction, two patients with ICH, and two patients with MS	Six with unilateral Gpi, one with bilateral Gpi, one with bilateral VIM, and two with unilateral VIM	64% mean modified TRS improvement	Mean length of followup: 5.8 years

Follett et al. (2014) [38]	Posttraumatic HT (symptoms for 15 years)	Bilateral VIM	Reduction of tremor from a score of 3 to a score of 1 in the right arm and from 3.5 to 0 in the left arm (TETRAS scale)	12 months

Grabska et al. (2014) [39]	Ischemic left thalamic stroke (symptoms 30 years)	Contralateral VOA and Zi	TRS 73% reduction in tremor	4 years

Kobayashi et al. (2014) [40]	1. Brainstem thalamus hemorrhage (symptoms for 6 years) 2. Cerebral infarction (symptoms for 3 years) 3. Intracerebral midbrain hemorrhage (symptoms 8 months) 4. Posttraumatic (symptoms for 2 years)	Four patients with dual-lead stimulation of ventralis oralis/ventralis intermedius nuclei (VO/VIM) and PSA	87% mean improvement in tremor	25 months

Castrop et al. (2013) [36]	1.Hypertensive mesencephalic hemorrhage (symptoms for 1 year) 2.pontomesencephalic AVM hemorrhage (symptoms for 2 years)	Contralateral VIM	Good tremor suppression, whereas the other symptoms remained unchanged	7 years and 6 years, respectively

Issar et al. (2013) [37]	One patient with posttraumatic tremor (symptoms for 6 months) with associated dystonia and cerebellar and cognitive difficulties.	Bilateral VIM	Partial benefit (CGI scale 3). No TRS available. Dystonia persisted	N/A

Aydin et al. (2013)	Midbrain and pons bleeded cavernoma	Right VIM + GPi	68% improvement in TRS left hand score	6 months

Acar et al. (2010) [35]	Subarachnoid hemorrhage (symptoms less than 1 month)	Bilateral VIM	No tremor and reduction in disability due to tremor	3 months

Sanborn et al. (2009)	Midbrain cystic degeneration	Right VIM	Full tremor suppression	2 years

Bandt et al. (2008) [31]	Left midbrain cerebral infarction (symptoms for 7 months)	Left lenticular fasciculus	Almost complete resolution of postural and intention tremors; scored 1/4 on the WHIGET	16 months

Diederich et al. (2008) [32]	1. L venous pontine angioma (symptoms for 7 years) 2. R midbrain hemiatrophy (symptoms for 32 years)	Contralateral VIM	Substantially ameliorated postural > rest > intention component	7 years and 5 years, respectively

Peker et al. (2008) [33]	Left thalamic abscess (symptoms 18 months)	Right VIM	90% overall improvement	2.5 years

Plaha et al. (2008) [34]	No obvious MRI abnormality (symptoms for 6 years)	Contralateral caudal Zi	70.2% improvement in total TRS	N/A

Lim et al. (2007)	Midbrain hemorrhage from a cavernous malformation (symptoms for 9 months)	Right VIM + VOA + GPi	7% improvement with GPi DBS and 0% with VIM or VOA DBS	8 months

Foote et al. (2006) [2]	Posttraumatic tremors: Three patients with symptoms for 16 years, 3 years, and 4 years	Two patients with VIM (border VIM/VOP and one with border VOA/VOP)	Total TRS improvement of 38.46 %, 48.33%, and 66.67 %, respectively	12 months, 6 months, and 8 months, respectively

Nikkhah et al. (2004) [29]	1.Right infarct midbrain (tremor symptoms 6 months); 2. Left thalamic AVM	Two patients with contralateral VIM	Almost complete tremor resolution (80% improvement). Dystonia and rigidity benefit reported	7 months and 6 months, respectively

Piette et al. (2004) [30]	Pontine tegmental hemorrhage	Right VIM	Major functional improvement	16 months

Romanelli et al. (2003) [27]	Unknown, severe symptoms 6 years	Left VIM and left STN	Tremor component improved 66%.	2 years

Samadani et al. (2003) [28]	Left midbrain cavernous malformation (symptoms for 4 years)	Contralateral VIM	57% increase in dexterity and four-point decrease in functional disability in TRS.	N/A

Pahwa et al. (2002) [26]	Midbrain cavernous hemangioma (symptoms for 3 years)	Right VIM	Significant improvement in postural and resting tremor; kinetic component persisted.	10 months


AVM = arteriovenous malformation, DBS = deep brain stimulation, DRTT = dentatorubrothalamic tract, GPi = globus pallidus, HT = Holmes tremor, NA= not assessed, TRS = Fahn–Tolosa–Marin tremor rating scale, Raprl = prelemniscal radiation, VIM = ventral intermediate nucleus, VOA = ventral oral anterior nucleus, VOP = ventral oral posterior nucleus, Zi = zona incerta.

GPi DBS has shown marked benefit in tremor, particularly in the resting tremor component in HT patients. In cases where the thalamus is severely damaged or in cases with associated dystonia or chorea, GPi DBS has been proposed as a potential target [4,5] (***Video 1***). In addition, neurostimulation of the posterior subthalamic area including the pre lemniscal radiations, caudal zona incerta, or motor thalamus (ventralis oralis anterior (VOA), and ventralis oralis posterior (VOP)) in combination with VIM DBS has been successful in single case reports or case series [5]. Recently, the use of multiple ipsilateral targets (2 or 3) including the thalamus and subthalamic area, the ventralis intermedius and subthalamic nuclei have been explored. Results have thus far been inconsistent and difficult to compare with single lead approaches [4,5,6,9,11,14,27,28]. Although clinical outcome scales vary among studies, the average overall improvement in tremor with DBS is 76% with an average age of 41 years (range 11 to 84 years), HT duration of 6 years (range 6 months to 32 years), and an average follow-up of 3 years (range 6 months to 12 years).

**Figure 2:**
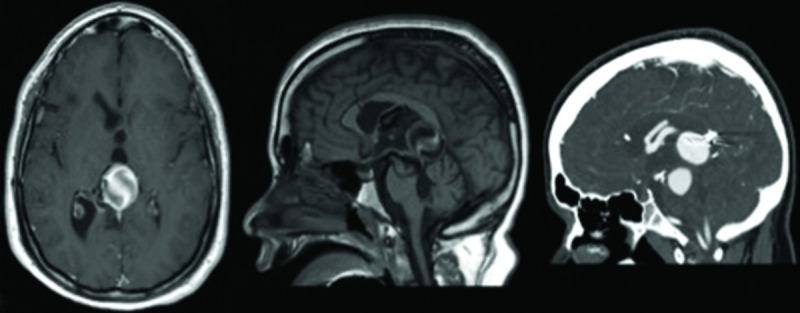
Axial (left) and sagittal (middle) T1 weighted MRI with gadolinium, and sagittal CT scan (right) of unsecured AV fistula.

**Video 1:** **Holmes tremor treated with unilateral Globus Pallidus Interna DBS.** Patient is a 24-year-old male who suffered a pontine and midbrain hemorrhage secondary to rupture of AV fistula at age 17. In addition, a large varix was found in the vein of the Galen and brainstem in the setting of a complex AV fistula (***Figure 2***). Associated neurological symptoms include ataxia, oculomotor difficulties, spastic dysarthria, dystonia and right arm clumsiness. Over the following 6 months, the patient noted insidious onset of progressive right arm low-frequency resting, postural, and action tremor diagnostic of HT. Patient underwent right globus pallidus internus DBS surgery. At six month follow up, tremor rating scales showed 80% overall improvement with marked improvement in daily activities.

Sustained positive DBS effects has been reported for up to nine years [6,28] but most data reported are relatively short term and reports of tremor recurrence over time are likely underreported [6,28]. Thus, continued optimization of DBS stimulation settings might be required [5]. Given the highly heterogenous nature of HT, diverse treatment options, and variable response to treatment, there is currently no consensus regarding HT management strategies [9]. Based on this systematic review of the literature, a reasonable algorithm for the treatment of HT is proposed here: Medical management with trihexyphenidyl, carbidopa-levodopa, and levetiracetam should be considered as first line treatment options for most patients and can be combined with other drugs including zonisamide, clonazepam, or bromocriptine. If benefit is limited or side effects limit medical management, DBS can be considered with lesional approaches considered second line. Target selection should be based on systematic review of the literature, a reasonable algorithm for the treatment of HT has been proposed [9].

## Discussion and recommendations

Our understanding of HT pathophysiology continues to evolve since its original description over 100 years ago. The rich and complex phenomenology of this rare tremor syndrome presents an important clinical challenge for physicians. Most of the available evidence regarding treatment and pathophysiology is limited to small case series or case reports with a lack of prospective and randomized studies. Furthermore, the variable nature and injury to multiple and/or different anatomical brain regions likely underlies the inconsistent response to medical and surgical interventions to date and highlights the need for a personalized management approach. Common etiologies for HT include vascular, traumatic, and demyelinating disorders affecting multiple brain networks including the dopaminergic nigrostriatal system, cerebello-thalamo-cortical pathway, and dentate-rubro-olivary pathway, creating abnormal neuronal and network activity. Nonetheless, the specific mechanisms underlying the development of HT remain poorly understood.

A detailed assessment of the associated neurological signs and tremor phenomenology including cerebellar findings, dystonic postures, chorea, or abnormal muscle tone can all be crucial for an appropriate diagnosis and treatment selection. Management remains challenging, with partial, transient, or lack of benefit noted by some patients with medical therapy leading to marked functional disability long term. The three most frequently used and published medications showing clear tremor benefit include carbidopa-levodopa, trihexyphenidyl, and levetiracetam and should be considered first-line treatment. Medication selection should be based on clinical and functional improvement, associated medical comorbidities and tolerability. Second line agents should be used in combination or as monotherapy following a similar clinical approach, despite the lack of controlled studies. In refractory cases, DBS should be considered as neuromodulation can effectively regulate the aberrant neuronal circuitry in HT. Target selection should be based on associated clinical characteristics, neuroimaging, and experience. Thalamic or pallidal stimulations have demonstrated good clinical outcomes. A recent systematic review suggests potentially better tremor and overall benefit with pallidal DBS. GPi DBS should be considered in HT patients with other associated hyperkinetic disorders including dystonia or chorea. Pallidal stimulation could be applied in patients with disrupted thalamic anatomy or structural brain lesions affecting the thalamic targeting.

Management of intention and proximal tremor component in HT has been particularly difficult with thalamic DBS, partially due to associated ataxia with larger levels of stimulation and a limited effect of thalamic stimulation for proximal limbs. Dual lead stimulation with VIM and VOA nucleus or other targets could be considered when the initial tremor benefit is limited or short lived. At our institute, this decision takes place intraoperatively and if VIM DBS fails to provide significant tremor control during macrostimulation, a VOA lead is placed approximately 2 mm anterior to VIM lead. Other targets stimulating the subthalamic area are being applied either in addition to VIM DBS with encouraging results [29]. Additional targets have also provided added benefit in tremor control. For example, targeting different tremor components with thalamic and subthalamic nucleus DBS, leads to a 66% improvement in tremor [30]. Despite these promising results, further well-designed, prospective, controlled studies are needed to: 1) validate the efficacy of these approaches, 2) better manage refractory HT, and 3) improve selection criteria for candidates. However, given the rarity of HT, it is unlikely that large, controlled trials would be feasible. Thus, collaborative efforts among institutions will be critical to advance research. Reporting associated surgical complications, stimulation side effects, and patients with suboptimal response or unresponsive cases will be critical to characterizing appropriate candidates, refining targets and approaches, and identifying the risks of therapy in HT. Advances in DBS technology will likely aid in the continued innovation and progress of DBS for HT. Increasing sensing capabilities might allow a refinement of the neurophysiological correlates and provide additional information to direct programming. Finally, it is possible that closed loop neuromodulation will be applied for complex cases with the potential to minimize side effects particularly when two leads are required for tremor control and in patients with associated cerebellar features, dysarthria, or gait difficulties.

In conclusion, medical and surgical therapies are viable strategies for the management of HT. The risks and benefits of each intervention should be carefully discussed with the patient and multidisciplinary team to improve outcomes. The selection of specific medication or DBS targets should be based on associated phenomenology, clinical features, neuroimaging, and treatment center experience.
